# Exploring Rosiglitazone’s Potential to Treat Alzheimer’s Disease through the Modulation of Brain-Derived Neurotrophic Factor

**DOI:** 10.3390/biology12071042

**Published:** 2023-07-24

**Authors:** Mackayla L. Nelson, Julia A. Pfeifer, Jordan P. Hickey, Andrila E. Collins, Bettina E. Kalisch

**Affiliations:** Department of Biomedical Sciences and Collaborative Specialization in Neuroscience Program, University of Guelph, Guelph, ON N1G 2W1, Canada; mackayla@uoguelph.ca (M.L.N.); pfeiferj@uoguelph.ca (J.A.P.); jhicke01@uoguelph.ca (J.P.H.); andrila@uoguelph.ca (A.E.C.)

**Keywords:** rosiglitazone, brain-derived neurotrophic factor, Alzheimer’s disease, clinical trials, peroxisome proliferator-activated receptor-gamma, type 2 diabetes mellitus, review

## Abstract

**Simple Summary:**

This review focuses on rosiglitazone, a medication used to treat diabetes. Rosiglitazone lowers blood sugar levels by helping the body use insulin more efficiently. Individuals with diabetes have a higher risk of developing Alzheimer’s disease, a complex memory-loss disorder affecting millions of individuals globally. Although scientists do not know why, diabetes may interfere with the ability of the brain to respond to insulin. Diabetes and Alzheimer’s disease have a lot in common when it comes to symptoms, brain changes and disease progression. As a result, researchers are investigating the potential of anti-diabetic drugs, such as rosiglitazone, to treat Alzheimer’s disease. Although the results in human clinical trials have not been promising, rosiglitazone provided significant improvements in cellular and animal models of Alzheimer’s disease, with even more promising results observed when rosiglitazone was formulated with nanosized particles that can assist with drug delivery. This review proposes that rosiglitazone may provide these benefits by modulating brain-derived neurotrophic factor, a critical protein for brain and metabolic health.

**Abstract:**

Alzheimer’s disease (AD) is a progressive neurodegenerative disorder that debilitates over 55 million individuals worldwide. Currently, treatments manage and alleviate its symptoms; however, there is still a need to find a therapy that prevents or halts disease progression. Since AD has been labeled as “type 3 diabetes” due to its similarity in pathological hallmarks, molecular pathways, and comorbidity with type 2 diabetes mellitus (T2DM), there is growing interest in using anti-diabetic drugs for its treatment. Rosiglitazone (RSG) is a peroxisome proliferator-activated receptor-gamma agonist that reduces hyperglycemia and hyperinsulinemia and improves insulin signaling. In cellular and rodent models of T2DM-associated cognitive decline and AD, RSG has been reported to improve cognitive impairment and reverse AD-like pathology; however, results from human clinical trials remain consistently unsuccessful. RSG has also been reported to modulate the expression of brain-derived neurotrophic factor (BDNF), a protein that regulates neuroplasticity and energy homeostasis and is implicated in both AD and T2DM. The present review investigates RSG’s limitations and potential therapeutic benefits in pre-clinical models of AD through its modulation of BDNF expression.

## 1. Introduction

Rosiglitazone (RSG) is a peroxisome proliferator-activated receptor-gamma (PPARγ) agonist of the thiazolidinedione (TZD) class used to treat type 2 diabetes mellitus (T2DM) and potentially Alzheimer’s disease (AD) [1,2,3,4,5]. T2DM and AD are known to share similar disease characteristics, most notably impaired insulin signaling and glucose metabolism [6,7,8,9]. These similarities, together with the limited number of AD treatments available, have prompted researchers to investigate the use of easily accessible anti-diabetic agents for AD treatment [10,11,12,13,14,15,16,17,18,19,20,21,22,23]. 

RSG has been observed to attenuate pathological hallmarks and behaviors associated with AD in pre-clinical in vitro and in vivo models; however, clinical trials investigating its efficacy in treating cognitive decline associated with AD and T2DM reported no significant improvements [3,4,5,10,11,12,13,14,15,16,17,18,19,20,21,22,23]. The discrepancies in results between pre-clinical and clinical studies, along with RSG’s low blood-brain barrier (BBB) permeability and cardiovascular risks, highlight its limitations [11,12,24,25,26]. Recent research exploring alternative drug deliveries, such as the encapsulation of RSG with nanoparticles, displays enhancements to both RSG’s BBB permeability and improvements in targeting the pathological hallmarks of AD [12,25,26]. These promising pre-clinical results, together with a better understanding of RSG’s disease-modifying mechanisms, may contribute to the modification or development of potential AD treatments. Although RSG may work through a variety of mechanisms, one that may be particularly relevant for improvements in pre-clinical AD models is RSG’s ability to modulate the expression of brain-derived neurotrophic factor (BDNF), its receptors and key signaling pathway modulators [12,25,26,27,28,29,30]. 

BDNF is a member of the neurotrophin family known for regulating a variety of neuronal and physiological processes [31,32,33,34,35,36]. Altered BDNF expression is observed in AD, and although data is lacking for the effects of RSG on BDNF in humans, in pre-clinical models of T2DM and AD, RSG upregulates BDNF and improves memory and cognition [12,25,26,27,28,29,30,31,32,33,34,35,36]. Considering BDNF’s role in neuronal functions together with RSG’s attenuation of AD-related pathologies and modulation of BDNF, this review explores the possibility that RSG’s benefits in pre-clinical models of AD are due at least in part to its ability to enhance BDNF expression and signaling.

## 2. General Features of BDNF

### 2.1. Expression and Isoforms

The *BDNF* gene is located on chromosome 11p14.1 and is transcriptionally regulated by many factors, the most well-known being cyclic AMP-response element binding protein (CREB) [37,38,39,40,41,42,43,44,45,46]. Interestingly, a bidirectional relationship exists between CREB and BDNF, such that both substrates are responsible for activating each other. Activated CREB promotes the transcription of BDNF by binding to the calcium response element in exon IV of BDNF’s promoter region, while BDNF activates CREB through several calcium-dependent mechanisms [41,42,43,44,45,46]. Activation of the tropomyosin receptor kinase B (TrkB) signaling cascade triggers an increase in intracellular calcium, which activates CREB kinases (CaMKs), which phosphorylate CREB at its Ser133 site to activate it [42,43,44,45,46]. Consequently, the expression and activity of either BDNF or CREB can impact the expression of the other [46]. This implication will be further discussed in the following sections.

Following transcription, BDNF protein is synthesized in the endoplasmic reticulum (ER) as preproBDNF, which is a 247-amino acid peptide composed of an N-terminal 18 amino acid signal peptide, a 118 amino acid pro-region and a C-terminal 110 amino acid mature domain [47,48,49,50,51,52,53]. In the ER, the signal peptide sequence of preproBDNF is cleaved by convertases to form proBDNF [52,53], which can remain in its immature form or be converted into its mature form, mBDNF, by intracellular convertases including furin and prohormone convertase 1–3, or by extracellular convertases such as plasmin and matrix metalloproteinases 2 and 9 [47,48,52,53,54]. Protein complexes, such as sortilin and carboxypeptidase-e facilitate the sorting and release of BDNF [55,56]. A more comprehensive analysis of BDNF’s synthesis, processing, and secretion can be found in reviews by Lu et al. and Lebmann and Brigadaski [52,53]. 

**Figure 1:**
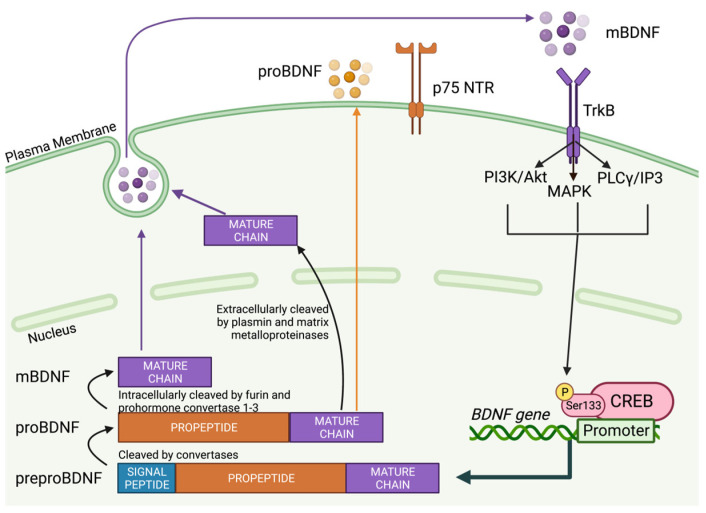
Schematic representation of brain-derived neurotrophic factor (BDNF) formation, processing, secretion and receptor activation. Created with BioRender.com (accessed on 30 April 2023).

Once proBDNF and mBDNF are synthesized, they can bind to their specific receptors and activate signaling pathways for their distinct biological functions [57,58]. proBDNF binds to the p75 neurotrophin receptor [p75 NTR] to activate pro-apoptotic and cytotoxic pathways, such as c-jun N-terminal kinase (JNK)/caspase 3, while mBDNF binds to TrkB to activate trophic signaling cascades, including phosphatidylinositol 3-kinase (PI3K)/Akt, phospholipase C (PLC)/inositol-1,4,5-trisphosphate (IP3) and mitogen-activated protein kinase (MAPK)/extracellular signal-regulated kinase (ERK)1/2 [31,32,45,58]. ProBDNF promotes apoptosis and long-term depression while decreasing the number and branching of neural dendritic trees, whereas mBDNF encourages neurogenesis, the growth of dendritic trees and long-term potentiation (LTP), processes known to contribute to learning and memory [53,55,59,60,61,62,63]. A schematic representation of BDNF’s processing, secretion and receptor activation is depicted in Figure 1.

### 2.2. Relevance, Limitations and Therapeutic Potential

In the central nervous system (CNS), BDNF is expressed by a variety of neuronal and glial cell types [31,32,33,64,65]. BDNF is highly concentrated in hippocampal, thalamic, and cortical regions of the brain, where it is known to play major roles in learning and memory, synaptogenesis, neurogenesis, synaptic plasticity, and neuroprotection [31,32,33,35,37,64,65]. Additionally, BDNF plays crucial roles in metabolic and physiological processes in other tissues and is expressed in the heart, gastrointestinal tract, skeletal muscle, adipose tissue, and platelet cells [32,66,67]. 

Given the broad distribution of BDNF throughout the body and its extensive range of functions, it is a frequently evaluated molecular target in metabolic and neurodegenerative diseases, including AD and diabetes, and for drug-targeting mechanisms as well, such as with RSG [12,25,26,31,32,33,34]. Since the prevalence of AD and T2DM is on the rise and, notably, BDNF expression undergoes significant alterations in these pathological conditions, the development of novel treatments or utilizing already available ones, such as RSG, that target BDNF, its receptors and key signaling pathways is necessary [11,31,32,33,34].

BDNF-centered therapies have been explored for the treatment of AD, with endogenous treatments including gene delivery, antidepressants, lifestyle changes and supplementation and exogenous treatments utilizing nanoparticle formulations [64,68]. As will be described for RSG later in this review, BDNF-based therapies show promising results in pre-clinical AD studies, but these benefits are not observed in clinical trials [64]. Translational concerns for BDNF include invasive routes of administration, unregulated dosage and degradability, poor systemic circulation, inability to cross the BBB and adverse side effects [64,69,70]. This highlights many of the common issues with drug design and development for AD, as the translatability of drugs in pre-clinical stages fails to provide any significant improvements in human clinical trials. 

To address these limitations, gene therapies and nanoparticle formulations are growing in popularity as they present better-targeted deliveries and effective results with minimal side effects in pre-clinical models and thus have the potential to be tested in clinical trials [64,69,70,71]. In February 2023, a 5-year-long phase 1 clinical trial of novel adenoviral AAV2-BDNF gene therapy was initiated to test its ability to decrease neuronal loss and promote synaptogenesis in patients with mild cognitive impairment (MCI) and AD [72]. Additionally, a recent liposome-targeting nanoparticle formulation of a BDNF viral vector gene therapy was reported to reduce adverse effects and plaque load in 6- and 9-month-old amyloid precursor protein (APP)/presenilin-1 (PSEN) mice [73]. Like BDNF, RSG also consistently demonstrated benefits in pre-clinical models but failed to provide any improvements in AD-related pathologies and behaviors in human clinical trials; however, nanoparticle formulations of commonly accessible diabetic drugs like RSG may represent a promising future direction for clinical trials [71].

## 3. Rosiglitazone: General Features of PPARγ and Treatment of Type 2 Diabetes Mellitus

T2DM is a metabolic disorder primarily characterized by chronic insulin resistance and hyperglycemia that impair pancreatic β-cell function and insulin secretion [1,74]. RSG is the most potent member of the TZD class of oral blood glucose-lowering medications known for their effectiveness in enhancing glycemic control and insulin secretion and decreasing blood glucose levels and insulin resistance [74]. PPARγ agonists regulate the expression of specific target genes that are dysregulated in a diabetic state [1,74,75,76]. One such target is the glucose transporter-4 (GLUT-4), which, under insulin-resistant conditions, is not translocated efficiently to the cell membrane, resulting in impaired insulin-regulated glucose uptake [75]. TZD-mediated activation of PPARγ enhances GLUT-4 transcription and cell surface expression, facilitating efficient glucose uptake and increased insulin sensitivity [75].

PPARγ is a ligand-activated transcription factor that, upon activation, heterodimerizes with the retinoid X receptor (RXR) to bind to peroxisome proliferator response elements (PPREs) in DNA promoter regions [76]. PPARγ is expressed in virtually all tissues. In the periphery, it is highly expressed in adipose tissue, skeletal muscle, and liver, and the CNS, it is expressed in neurons, astrocytes, and oligodendrocytes across several brain regions, such as the prefrontal cortex, hippocampus, nucleus accumbens and amygdala [1,77,78,79]. PPARγ is known for its activation of anti-inflammatory and antioxidant pathways, but in the CNS, it additionally promotes the growth of neural stem cells and the differentiation of neurons and oligodendrocytes [77,78,79]. Therefore, PPARγ agonists like RSG have been proposed as treatments for neurodegenerative diseases such as AD, where there is widespread neuron loss [77,78,79].

### 3.1. Pharmacokinetics

Following the administration of a 2 mg oral dose, RSG is rapidly absorbed from the gastrointestinal tract; the bioavailability of the compound is near complete at around 99%, with peak plasma concentration occurring at 1.3 h in a fasted state, compared to 3.5 h in a fed state [1,75]. Once absorbed, RSG is extensively distributed throughout the body due to its highly protein-bound state (over 99%), primarily to albumin, which is thought to contribute to its high volume of distribution into peripheral tissues [75]. The BBB exhibits extremely low permeability to RSG in rodents (0.045%) following intravenous (IV) administration, and it is suspected that this is similar in humans, although this contention has not been entirely elucidated [24,80]. RSG is primarily metabolized by the liver, specifically the CYP2C8 enzyme, and its metabolism results in the formation of active and inactive metabolites, N-desmethyl-RSG and hydroxy-RSG [75]. The half-life of RSG is estimated to be approximately 3–4 h in both fasted and fed states, and excretion occurs primarily via the kidneys [1,75]. Overall, RSG displays linear pharmacokinetic activity in doses ranging from 0.2 to 20 mg [75].

### 3.2. Adverse Effects

In the last decade, evidence has highlighted both cardiovascular and adverse effect risks associated with RSG treatment for prediabetes or T2DM [23,81,82]. Primary investigations and meta-analyses have raised concerns regarding RSG’s safety and its impact on cardiovascular disease (CVD) risk, including myocardial infarction, heart failure, and stroke [23,81,82]. The Food and Drug Administration (FDA) added a warning for RSG treatment in 2007, yet it remains an approved treatment based on the risk-benefit ratio, despite RSG being removed from the European market in 2010 due to concerns about its potential cardiac event risk [23,83,84,85].

Additionally, RSG has been linked to dose-dependent weight gain, fluid retention, edema, and a heightened risk of fracture [23,86,87,88]. RSG is not alone in displaying an increased risk of adverse events, other TZDs and diabetic agents elicit similar adverse effects as well [23]. To mitigate this, physicians should limit their use in certain at-risk populations, such as post-menopausal women at risk of developing osteoporosis or those with or at risk of developing CVD [81,82]. Nevertheless, these risks must be considered when investigating its potential therapeutic use in other conditions. Although RSG is typically used to treat T2DM, given the strong comorbidity and shared pathways between T2DM and AD, there is a compelling interest in investigating its use in AD.

## 4. Rosiglitazone and Alzheimer’s Disease

Dysregulated insulin signaling mechanisms have emerged as critical contributors to the development and progression of AD, prompting interest in the use of insulin-sensitizing agents, such as RSG, for managing AD-related insulin dysfunction [89,90,91]. This section will discuss the current understanding of RSG’s effects in preclinical models and human clinical trials of AD, focusing on its potential to enhance cognitive function and modulate pathophysiological mechanisms associated with AD.

### 4.1. Rosiligtazone’s Treatment of Alzheimer’s Disease-Related Pathology in Pre-Clinical Models 

#### 4.1.1. Cognitive Function

RSG was reported to improve cognitive function in preclinical models of AD. Numerous in vivo studies have demonstrated that RSG treatment significantly enhances learning and memory performance [13,14,16,17,92,93,94,95]. For example, Cortez et al. reported that 9-month-old transgenic (Tg2576) mice orally treated with 30 mg/kg of RSG displayed significant improvements in hippocampal neurocircuitry, which were associated with improvements in hippocampus-dependent spatial memory and associative fear memory [17]. Similarly, 3 mg/kg of oral RSG treatment was observed to improve spatial memory performance in a 4-month-old APPswe/PSEN [delta]E9 double transgenic (2xTg) mouse model, with RSG-treated groups displaying greater memory flexibility and alleviating spatial memory impairments induced by beta-amyloid (A*β*) [16]. However, some studies report RSG’s ineffectiveness in modulating specific cognitive impairments, specifically spatial reference memory and object recognition [15,96].

Furthermore, the oral administration of RSG has been suggested to alleviate cognitive deficits in AD mouse models by ameliorating synaptic dysfunction as well as reducing neuroinflammation [15,16,96]. Notably, RSG reduced glial fibrillary acidic protein (GFAP) staining in a 10-month-old triple transgenic (3xTg)-AD mouse model following the dietary administration of 50 mg/kg of RSG for 4 months and diminished the astroglial inflammatory reaction and GFAP intensity in a 4-month-old 2xTg-mouse model following 3 months of RSG administration [15,16]. Similarly, direct injection of RSG into the dentate gyrus at doses of 0.5 μM, 5 μM or 20 μM prevented the increase in pro-inflammatory markers, such as interleukin-1*β* and interferon-γ in Wistar rats intracranially injected with the 42 amino acid A*β* oligomer (A*β*1-42) [92].

Notably, numerous studies initiate RSG administration in transgenic mouse models of AD after the expected onset of major pathological changes in respective rodent species [13,14,15,16,17,19,94,97].

#### 4.1.2. Glycogen Synthase Kinase 3 Beta and Tau

RSG also influences additional AD-related signaling mechanisms that may contribute to its ability to enhance cognitive performance in preclinical models. The inhibition of glycogen synthase kinase 3 beta (GSK3*β*) has consistently provided neuroprotection across varying animal models of AD, with a common protective effect against tau pathologies, as reviewed by Avila and colleagues [98]. Evidence implicates GSK3*β* as a major mediator of tau phosphorylation, a protein with an imperative role in microtubule stabilization in the CNS [98]. In its hyperphosphorylated state, tau forms neurofibrillary tangles (NFTs), one of the major pathological hallmarks of AD and contributors to cognitive decline [99]. GSK3*β* can phosphorylate tau at multiple sites, promoting its hyperphosphorylation and thus aggregation, which is why its inhibition remains a therapeutic strategy for AD [98].

Studies employing AD animal models observed increased GSK3*β* inhibition following the oral administration of RSG [16,19]. Toledo and Inestrosa noted that RSG-mediated GSK3*β* inhibition in the hippocampus of 2xTg-AD mice facilitated the recovery of downstream Wnt signaling proteins *β*-catenin and Dvl-3, two important regulators of the cell cycle [16]. In the context of AD, impairment of Wnt signaling by activation of GSK3*β* increases the production of AD pathologies and reduces cognition [100]. In addition to reduced GSK3*β* activity and recovered Wnt signaling, 12-week oral administration of RSG significantly decreased A*β* levels in the hippocampus of 3xTg-AD mice [16].

The ability of RSG to inhibit tau phosphorylation has been reported in various animal and cellular studies [14,15,94,98,101]. In animal models of AD, including transgenic mice expressing multiple APP mutations [14], 3xTg-AD mice [15], and spontaneously diabetic (OLETF) rats injected with streptozotocin (STZ) [98], decreased tau phosphorylation was observed after oral administration of RSG. Similarly, in SH-SY5Y human neuroblastoma cells, RSG treatment ameliorated bisphenol A (BPA)-induced toxicity [101]. Exposure to BPA is known to inhibit insulin signaling and increase levels of A*β* and hyperphosphorylated tau, and these effects were attenuated in SH-SY5Y cells treated with RSG [101].

#### 4.1.3. Amyloid-Beta

A*β*, a peptide derived from the cleavage of APP, plays a central role in AD pathogenesis, and when present in excess quantities, it can aggregate to form A*β* plaques [102]. Accumulation of A*β* in the brain is linked to inflammation, synaptic dysfunction, and neuronal death [102].

RSG has been observed to significantly modulate A*β* levels in various experimental models of AD [14,16,94,95,103,104]. An in vitro study by Wang and colleagues determined that RSG attenuated the BPA-induced increase in APP, beta-site APP cleaving enzyme 1 (BACE1), and A*β*1-42, all of which are key proteins involved in the pathogenesis of AD [101]. Notably, BACE1 together with γ-secretase cleaves APP to generate A*β* peptides, including the A*β*1-42 peptide, which is prone to aggregation [20,102]. Additional information regarding the role of numerous proteins contributing to AD, including APP, BACE1, and A*β*1-42, can be found in the 2016 review by Selkoe and Hardy [102].

Chiang et al. observed a decrease in A*β* levels in human neural stem cells treated with RSG [18]. This was attributed to the RSG-mediated downregulation of caspase 3 and 9 activity, both of which were reported to be increased and linked to excessive cellular death in pathological conditions like AD [18]. In vivo studies have similarly reported reduced hippocampal A*β* levels in AD mouse models following RSG treatment [13,14,94,95]. Li et al. investigated the effects of RSG on insulin-degrading enzyme (IDE) and APP in 4-month-old APPswe/PSEN mice injected with STZ to induce diabetes [95]. In addition to metabolizing insulin, IDE degrades A*β*, and reduced expression of IDE has been associated with A*β* accumulation and plaque formation [105,106]. Li et al. observed that subcutaneous injection of 50 mg/kg RSG increased IDE levels, which was followed by a significant reduction in A*β* levels, demonstrating RSG’s capacity to modulate the pathological mechanisms associated with AD [95].

As previously outlined, various studies have demonstrated the crucial role the PPARγ pathway plays in RSG’s effects [97,101,103,107]. Wang et al. observed a reduction in APP expression and A*β* secretion along with an increase in IDE expression and A*β* degradation in RSG-treated SH-SY5Y cells [108]. These effects were blocked when cells were treated with the PPARγ antagonist GW9662. These findings are consistent with Camacho et al., who demonstrated that PPARγ activation enhanced brain A*β* clearance mechanisms [109]. Moreover, RSG treatment prevented A*β*-induced toxicity in rat hippocampal neurons, an effect reversed by the PPARγ inhibitor FH353, again suggesting PPARγ activation was necessary for RSG’s neuroprotection [103]. Lastly, dietary administration of 30 mg/kg RSG facilitated the convergence between PPARγ and ERK signaling in an 8-month-old Tg2576 AD mouse model, with ERK signaling being essential for hippocampal-dependent learning and memory in rodents [97]. Therefore, RSG acts through PPARγ to modulate the amyloidogenic pathway.

Despite low BBB permeability, RSG demonstrates therapeutic potential in pre-clinical models of AD via the enhancement of multiple signaling mechanisms, improvement in cognitive and synaptic function, reduction of neuroinflammation, and modulation of key signaling proteins involved in the pathogenesis of AD, such as GSK3*β*, tau, and A*β*. This prompted the exploration of RSG’s efficacy in human clinical trials.

### 4.2. Rosiglitazone in Alzheimer’s Disease Clinical Trials

Table 1 summarizes the data from human clinical trials investigating RSG in AD [5,20,110,111,112,113]. This summary includes results published within the last two decades and those available from ongoing clinical trials. Data from clinical trials were collected from the NIH U.S. National Library of Medicine site: ClinicalTrials.gov. The inclusion criteria for the clinical trials discussed in this review required that the study (1) included participants diagnosed with a neurodegenerative condition with evidence of cognitive decline, such as AD, (2) utilized RSG as the only form of treatment for AD, (3) utilized RSG in conjunction with standard drugs commonly used to treat AD, such as acetylcholinesterase inhibitors (AChEIs) like donepezil and (4) studies that provided results.

Multiple clinical trials assessed the safety of RSG and its effects on cognition and pathological markers in patients with AD [5,20,110,111,112,113]. In these studies, 2 mg, 4 mg, 8 mg, or 10 mg doses of extended-release (XR) RSG were administered orally to AD patients for various durations (24 to 54 weeks). Although results from a preliminary trial demonstrated improvements in delayed recall and selective attention in AD patients receiving 4 mg of RSG daily for 6 months, additional trials reported that RSG XR alone and as an adjunct therapy did not significantly improve clinical outcomes, functional brain activity or cognitive function in AD patients [20,111,112,113]. Additionally, concerns regarding the safety and tolerability of RSG XR also arose, as one study reported that although RSG XR did not induce severe or life-threatening events, one-third of the participants experienced adverse events [110].

To assess the change from baseline in global and regional indices of cerebral metabolic rate, participants received either a placebo or 4 mg of RSG XR tablets once daily (OD) for 1 month, increasing to 8 mg OD for a total of 12 months. The results suggested that although RSG was associated with an early increase in the metabolism of whole brain or global glucose, there was no clinical or biological evidence to support the ability of RSG to slow disease progression in the symptomatic stages of AD [5]. This negative finding was also consistently observed in trials in which the effects of RSG XR alone were compared to those of donepezil or placebo as monotherapy on cognition and overall clinical response in participants with mild to moderate AD. The primary outcome measure was to assess the change from baseline in mean Alzheimer’s Disease Assessment Scale-Cognitive subscale (ADAS-Cog) total score and mean Clinician’s Interview-Based Impression of Change plus caregiver input (CIBIC+) global functioning total score at the 24th week, and participants were stratified into apolipoprotein e4-positive and e4-negative groups and randomized 2:2:2:1 to OD doses of placebo, 2 mg RSG XR, 8 mg RSG XR or 10 mg donepezil (control) [111]. RSG XR monotherapy did not improve cognition or global function in any of the analysis populations. Another study that aimed to assess the change from baseline in ADAS-Cog total score and Clinical Dementia Rating Scale-Sum of Boxes (CDR-SB) at the 48th week similarly found that there was no statistical or clinical difference in the efficacy of 2 mg or 8 mg RSG XR over 54 weeks as a form of adjunctive therapy with AChEIs on cognition and global function in patients with AD [112,113].

The doses of RSG used in AD clinical trials are consistent with those used for T2DM, with therapeutic doses ranging from 2 mg to 8 mg [3,114,115,116,117,118,119,120,121]. Although these doses improve insulin sensitivity and lower glucose levels, the currently available data for human clinical trials do not provide support for the role of RSG as a treatment for AD [3,115,116,117,118,119,120,121,122]. Possible factors contributing to the failure of RSG in human clinical trials include species differences in AD-like disease progression and the action of RSG and its PPARγ mechanism, the low BBB permeability of RSG, as well as the recruitment of suboptimal or wrong target groups in clinical trials [24,80]. It is now clear that interventions that target patients with AD have proven to be ineffective due to the degree of disease progression from excessive amyloidogenesis and tau hyperphosphorylation [122]. Thus, clinical trials should target groups with earlier stages of mild cognitive decline. 

Despite its failure in human clinical trials, the observed benefits of RSG in preclinical models of AD, together with the abundance of data supporting the role of impaired brain insulin signaling in AD pathology, suggest anti-diabetic drugs should be further investigated as a therapeutic option for AD. In addition, recent advances in drug delivery, such as nanoencapsulation, represent promising strategies for future human clinical trials [71,73]. Nanoparticle formulations of easily accessible drugs like RSG could improve drug transport through the BBB, increase drug concentrations in the target area of interest and lower the risk of adverse effects [71,73,123]. Additionally, a better understanding of the mechanisms of anti-diabetic drugs like RSG may identify therapeutic targets that could be investigated in future clinical trials. Recently, RSG formulated with nanoparticles was shown to modulate BDNF and its upstream signaling pathway substrates in models of diabetes and AD [12,25,26,27,28]. To better understand how RSG may act through this mechanism, alterations to BDNF expression and its pathological involvement in AD must be explored.

## 5. BDNF and Alzheimer’s Disease

BDNF levels are known to fluctuate in response to factors such as age, sex hormones, lifestyle, and stress; however, chronic alterations in its expression can be indicative of or potentially lead to chronic diseases, such as AD [31,32,33,124,125,126].

Although studies generally suggest increasing BDNF levels and signaling would be beneficial for multiple neurological disorders, conflicting trends in BDNF levels have been reported in AD [31,33,127]. Key studies by Faria et al., Laske et al. and Ng et al. reported increased BDNF plasma and serum levels in the early stages of AD and MCI; however, levels of BDNF have been reported to decrease with elevated levels of A*β*, GSK3*β*, tau and cortisol in the later stages of AD progression [33,34,35,36,64,70,128,129,130,131,132,133,134,135,136,137,138]. Additionally, decreased BDNF expression has also been associated with atrophy in the hippocampus, medial temporal lobe, and neocortex, which are regions that are also known to be significantly implicated in AD pathology [50]. Furthermore, studies focusing on AD as “type 3 diabetes” reported decreased levels of BDNF in individuals with T2DM and MCI/dementia [139,140,141,142]. These findings, in conjunction with in vitro and in vivo pre-clinical studies, have propelled researchers to speculate that early in the AD disease process, BDNF expression may be upregulated in a “last attempt” to rescue the brain from AD pathology; however, as the disease progresses, the widespread pathology and neurodegeneration associated with AD may ultimately result in the decreased expression of BDNF [31,70,128,129,130,131,132,133,134]. 

Ng et al. and others proposed that the reported discrepancies in the levels of BDNF could also be due to differences in study methods, including the lack of a gold standard protocol for BDNF extraction and quantification and confounding factors such as the disease stage, current medications, psychiatric comorbidities, and collection of plasma of the patients [128,143,144]. In response to the validity of measuring BDNF in serum and plasma, they proposed that (1) platelet-free samples should be collected as platelets are known to alter BDNF levels, and (2) both proBDNF and mBDNF should be quantified as they have different biological functions and effects [128,143,144]. Although most studies report reduced BDNF expression in a neurodegenerative state, further studies are needed to better understand the reported discrepancies.

To better understand these fluctuations in expression, the pathological role of BDNF, specifically proBDNF, in the development of AD through the activation of inflammatory and apoptotic pathways has been explored. Chen et al. reported that accumulated proBDNF correlated with enhanced Aβ deposition, plaque formation and learning and memory deficits in a 2xTg mouse model of AD [145]. Wang et al. determined that BDNF depletion can contribute to this through increased inflammatory cytokines and activation of the Janus kinase2/signal transducer and activator of transcription protein3 (JAK2/STAT3) pathway, which leads to neuronal loss caused by APP and tau fragmentation [146]. Additionally, Fleitas et al. reported upregulation of proBDNF and sortilin in the hippocampus and a higher ratio of proBDNF to mBDNF in the cerebrospinal fluid of patients with AD through a gain-of-function mutation in the PSEN1 gene that may enhance proBDNF-induced cell death [147]. Considering that these mechanisms and effects have been similarly observed in rodent models of aging, there is the possibility that increased and accumulated levels of proBDNF during aging may predispose an individual to develop AD [148,149]. As a result, there has been a call to develop diagnostic techniques that measure the ratio of proBDNF to mBDNF in individuals at high risk of developing AD [150].

Furthermore, decreased BDNF levels may be the result of altered expression of its upstream and downstream targets, including CREB and TrkB. Previously, the bi-directional relationship between BDNF and CREB was described, and in AD, accumulation of A*β* and hyperactivation of GSK3*β* were reported to decrease the phosphorylation and activation of CREB in brain tissue collected from patients with AD, as well as in in vitro and in vivo models of AD, and thus we speculate that this may be a potential mechanism by which BDNF is downregulated [68,151,152,153]. Additionally, the overexpression of truncated TrkB, known as TrkB.T1 may be another mechanism through which BDNF expression is downregulated in AD [154]. This isoform is a dominant-negative inhibitor for TrkB, such that it inhibits full-length TrkB and regulates endogenous levels of BDNF [154]. Interestingly, in AD, TrkB.T1 levels increase while BDNF and full-length TrkB levels in the frontal cortex and hippocampus decrease, and these elevated levels of TrkB.T1 also correlate with memory and cognitive impairments in transgenic mouse models of AD [65,132,154]. The impact of this TrkB isoform is extensively reviewed by Tessarollo and Yanpallewar [154]. Since BDNF is largely regarded for its neuroprotective and neurotrophic properties, there is a compelling interest in determining whether the benefits of RSG in pre-clinical models may be due to its ability to regulate BDNF activity.

## 6. Rosiglitazone Modulates CREB, BDNF and TrkB Expression

Several studies have reported that RSG alters the levels of CREB, BDNF and TrkB. CREB is one of the main transcriptional activators of BDNF, and in response to RSG treatment, its expression increases. Watson et al. observed this in STZ-induced diabetic rats, where CREB activation was increased in rats treated with 20 mg/kg/day of RSG through oral gavage for 8 weeks [29]. They postulated that since signaling pathways such as Akt, p38 MAPK and PI3K are known to phosphorylate and activate CREB at Ser133, RSG may modulate CREB activity through activation of these pathways [29]. Kim et al. confirmed this by demonstrating increased phosphorylated levels of CREB, calcium calmodulin-dependent kinase II (CaMKII) and Akt in rat insulinoma (INS)-1 cells treated with RSG for 24 h [30]. Although they deduced that these upregulations were the result of a g-protein coupled receptor 40-PPARγ dependent mechanism activated by RSG, these results signify that RSG upregulates key signaling substrates involved in the activation of CREB [30]. Similarly, Zhao et al. determined that RSG upregulated the phosphorylation and activation of the insulin receptor substrate 1/Akt/CREB pathway to induce anti-inflammatory, anti-apoptotic and antidepressant-like effects in a mouse model of unpredictable chronic mild stress and primary neuronal and astrocytic cultures [155]. Since RSG increases the phosphorylation of CREB and its upstream signaling substrates to induce therapeutic effects across several disease models and neuronal cell types, this may then present as a mechanism for the upregulation of the expression of BDNF and one of its main receptors, TrkB. 

Sarathal et al. reported that RSG alone and when co-encapsulated as a nanoformulation upregulated CREB, BDNF, nerve growth factor (NGF) and glial cell-derived neurotrophic factor (GDNF) expression in both in vitro and in vivo mouse models of AD [12,25,26]. Similarly, Patel et al. observed that when either *Urtica dioica* leaf extract or RSG was administered orally to STZ-treated mice, the mRNA expression of hippocampal BDNF, TrkB and cyclin D1 was increased [156].

**Figure 2:**
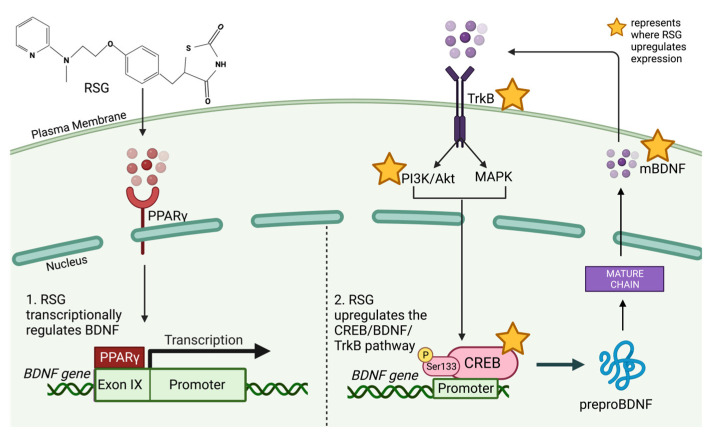
Rosiglitazone (RSG) modulating the expression of neural brain-derived neurotrophic factor (BDNF) through [1] peroxisome proliferator-activated receptor-gamma (PPARγ)-mediated transcriptional regulation and [2] upregulation of the cyclic AMP-response element binding protein (CREB)/BDNF/tropomyosin receptor kinase B (TrkB) pathway. Created with BioRender.com (accessed 30 April 2023, license agreement: KA25B75AY7).

Kariharan et al. reported improved cognition, enhanced LTP and significant increases in BDNF mRNA and protein expression by 3-fold and 2-fold, respectively, as well as additional upregulations in CREB and the N-methyl-D-aspartate (NMDA) and α-amino-3-hydroxy-5-methyl-4-isoxazolepropionic acid (AMPA) subtypes of glutamate receptors following intracerebroventricular (ICV)-administered RSG in diabetic mice [27]. Promoter analysis in transfected rat H19-7 hippocampal cells revealed 24-h RSG treatment increased BDNF transcription through PPARγ-mediated activation at exon IX of its promoter region, which contains a stop translation codon and encodes for full-length proBDNF [27,157]. The authors proposed that because BDNF regulates LTP through AMPA and NMDA receptors, and they reported that RSG increased protein and mRNA expression of these receptors as well as CREB and BDNF expression, this may present an additional mechanism by which RSG acts through BDNF to promote memory and cognition [27,158]. Additionally, Baghcheghi et al. administered RSG at doses of 2 and 4 mg/kg intraperitoneally for 6 weeks to propylthiouracil-induced hypothyroid rats [28]. Before RSG treatment, rats displayed decreased memory and cognitive performance, as well as reduced levels of BDNF; however, treatment with RSG improved performance in memory and cognition tasks, upregulated hippocampal BDNF levels and increased serum thyroxine levels, with no reported significant difference between the two different doses [28]. Although these studies did not use models of AD, there is the potential for RSG to treat other neurological and metabolic diseases through the modulation of BDNF. Figure 2 depicts the proposed mechanism through which RSG enhances neuronal BDNF levels.

These results highlight RSG’s efficacy in modulating BDNF, CREB and TrkB through oral routes of administration in in vivo models. However, an important limitation is the applicability of a rodent model as (1) the regulatory mechanisms controlling the *BDNF* gene, (2) the number of promoter and exon regions, (3) mechanisms generating BDNF transcripts, and (4) expression of BDNF and exon regions throughout several brain regions are different between rodents and humans, as outlined by Gao et al. [64]. Thus, there is a need to confirm many of these results by utilizing higher-order species or brain tissue samples collected from individuals with AD.

Additionally, these studies do not report whether the alterations in BDNF are to its pro or mature forms. As discussed by Ng et al., proBDNF and mBDNF activate distinct receptors and signaling pathways that are linked with specific functions [128,143,144]. To better understand the therapeutic potential of RSG, future studies must investigate whether RSG preferentially alters one of these forms of BDNF and determine the impact of this on other molecular outcomes of AD-related pathology.

### Rosiglitazone’s Modulation of BDNF for the Treatment of Alzheimer’s Disease

The effectiveness of RSG as a potential treatment for AD has mainly focused on its suppression of inflammatory genes, transcription factors and molecular pathways [159,160,161,162,163]. Since both BDNF and RSG are known to regulate neuronal function, and RSG modulates CREB, BDNF and TrkB expression in several disease models, we suggest that BDNF is another mechanism through which RSG may act. 

To date, Sarathal et al. are the only group that has investigated the effects of RSG on BDNF expression in vitro using SH-SY5Y cells and in vivo using an STZ-induced mouse model of AD [12,25,26]. Their first study compared the neuroprotective potential of RSG free form to the drug in the polyethylene (PEG)-polycaprolactone (PCL) polymer nanoformulated delivery system [12]. Following dose optimization and treatment, STZ-animals receiving RSG embedded into a nanocarrier system through oral, IV and ICV routes exhibited significant improvements in learning performance and memory retention, along with significantly reduced AChE activity, at lower doses than those treated with free-form RSG [12]. 

This study also revealed that RSG treatment significantly upregulated BDNF mRNA expression, along with other substrates, such as CREB, GDNF, NGF and PPARγ in both the hippocampus of mice and in SH-SY5Y cells [12]. RSG in free form at 10 mg/kg, 20 mg/kg, and nanoformulated at 5 mg/kg also increased neuronal density in the CA1 region of the hippocampus, improved neuron morphology and restored antioxidant levels in an ICV-STZ induced mouse model of AD. Based on these results, Sarathlal et al. concluded that (1) RSG upregulates neurotrophic factors like BDNF and improves memory and cognition in an AD-mouse model, (2) a nanoformulated carrier system offers neuroprotection at lower doses compared to the free drug form, and (3) RSG treatment can lower AChE activity, supporting the drug as a potential AD therapeutic [12].

These results prompted a follow-up study investigating the neuroprotective potential of RSG in combination with vorinostat, an epigenetic modulator, utilizing the same nanocarrier delivery system and STZ-induced mouse model of AD [26]. RSG in free form, RSG combined with vorinostat and RSG+vorinostat with the nanoparticle delivery system were administered both orally and IV for three weeks [26]. Although RSG in combination with vorinostat attenuated behavioral deficits associated with ICV-STZ induction, upregulated neurotrophic factor expression and reduced parameters of oxidative stress, the nanoparticle-encapsulated RSG elicited the greatest effect [26]. Compared to control wild-type mice, CREB and BDNF expression increased 3-fold in the oral and IV nanoparticle-treated groups, whereas RSG in combination with vorinostat elicited ~1.5-fold increase [26]. Similarly, a study utilizing an in vitro model of atherosclerosis reported that RSG combined with polylactic acid (PLA)-PEG polymer nanoparticles resulted in conserved safety, increased uptake and a 5-fold increase in efficacy [164]. Therefore, RSG delivered using a nanocarrier system has the potential to significantly boost drug delivery, efficacy, and neuroprotection, as demonstrated by increases in endogenous BDNF expression within a model of AD. 

Due to RSG’s safety concerns and its failure in AD clinical trials, there is a lack of information on its ability to modulate BDNF in this disease; however, the recent research conducted by Sarathal and colleagues highlights the potential of targeting BDNF and delivering drugs like RSG in nanoparticle formulations in future AD clinical trials.

## 7. Conclusions

Pre-clinical studies support the role of RSG as a treatment for AD, as demonstrated through improvements in AD-related symptoms and pathologies and increases in the expression levels of CREB, BDNF and TrkB. The benefits observed in these preclinical models, together with an abundance of data supporting the role of impaired brain insulin signaling in AD pathology, suggest that the modification of insulin sensitivity with compounds like RSG could provide therapeutic benefits.

To date, the results of human clinical trials have not supported the use of RSG as a treatment for AD. Unfortunately, this lack of success is a typical outcome for AD clinical trials [165]. A better understanding of the underlying mechanisms of AD pathology and symptoms, the contributions of other physiological systems to disease onset and progression, more effective biomarkers, the identification of multi-target drugs, and improved drug delivery systems could contribute to improved therapeutic approaches. Preclinical studies demonstrating increased effectiveness with RSG encapsulated within nanoparticles provide the rationale for exploring alternative drug delivery methods in clinical trials. Future research should investigate RSG’s molecular mechanisms to better understand the interplay between insulin sensitivity, PPARγ signaling and neuroprotection. 

## Figures and Tables

**Table 1:** Summary of clinical trials investigating RSG administration as a form of therapy in AD. RSG: Rosiglitazone; XR: Extended Release; ADAS-Cog: Alzheimer’s Disease Assessment Scale-Cognitive subscale; CIBIC+: Clinician’s Interview-Based Impression of Change plus caregiver input; CDR-SB: Clinical Dementia Rating Scale—Sum of Boxes; AChEI: Acetylcholinesterase Inhibitors.

Participants	Length of RSG Treatment	Primary Outcome Measures	Main Results	In-Text Reference
30 participants with AD or amnestic mild cognitive impairment	4 mg of RSG or placebo daily for 6 months	To assess cognitive performance and plasma Aβ levels	Participants that received RSG exhibited better delayed recall and selective attentive relative to participants that received placebo. Plasma Aβ levels were unchanged compared to baseline in participants that received RSG	[20]
33 participants with mild to moderate AD	4 mg of RSG XR orally, once daily for 4 weeks followed by 8 mg of RSG orally, once daily for 44 weeks	To assess the number of participants with adverse events	2 of 33 participants experienced serious adverse events while 10/33 participants experienced non-serious adverse events	[110]
80 participants with mild to moderate AD	4 mg of RSG XR once a day for 1 month increasing to 8 mg once a day or placebo for 12 months	Change from baseline in global and regional indices of the cerebral metabolic rate of glucose	Suggests that RSG is associated with an early increase in whole brain glucose metabolism but not any biological or clinical evidence for slowing the progression of AD	[5]
693 participants with mild to moderate AD	Once daily of placebo, 2 mg RSG XR, 8 mg RSG XR or 10 mg donepezil (control) for 24 weeks	To assess the change from baseline to week 24 in the ADAS-Cog score and CIBIC+ global functioning score	No evidence of 2 mg or 8 mg RSG XR monotherapy in cognition or global function	[111]
1496 participants with mild to moderate AD	Once daily of placebo + donepezil, 2 mg RSG XR + donepezil or 8 mg RSG XR + donepezil for 54 weeks	To assess the change from baseline to week 48 in ADAS-Cog and CDR-SB with the use of RSG XR as adjunctive therapy with donepezil treatment in AD	No evidence of statistically or clinically significant efficacy in cognition or global function was detected for 2 mg or 8 mg RSG XR as adjunctive therapy to ongoing AChEIs	[112]
1468 participants with mild to moderate AD	Once daily of placebo, 2 mg RSG XR or 8 mg RSG XR for 54 weeks	To assess the change from baseline to week 48 in ADAS-Cog and CDR-SB with the use of RSG XR as adjunctive therapy with AChEI treatment in AD	No evidence of statistically or clinically significant efficacy in cognition or global function was detected for 2 mg or 8 mg RSG XR as adjunctive therapy to ongoing AChEIs	[113]

## Data Availability

Not applicable.
